# Cognitive Aging and the Primate Basal Forebrain Revisited: Disproportionate GABAergic Vulnerability Revealed

**DOI:** 10.1523/JNEUROSCI.0456-23.2023

**Published:** 2023-12-06

**Authors:** Cristina Bañuelos, Joshua R. Kittleson, Katherine H. LaNasa, Christina S. Galiano, Stephanie M. Roth, Evelyn J. Perez, Jeffrey M. Long, Mary T. Roberts, Sania Fong, Peter R. Rapp

**Affiliations:** ^1^Laboratory of Behavioral Neuroscience, National Institute on Aging, Baltimore, Maryland 21224; ^2^California National Primate Research Center, University of California, Davis, Davis, California 95616

**Keywords:** basal forebrain, cholinergic, E/I balance, GABAergic, neurocognitive aging, nonhuman primate

## Abstract

Basal forebrain (BF) projections to the hippocampus and cortex are anatomically positioned to influence a broad range of cognitive capacities that are known to decline in normal aging, including executive function and memory. Although a long history of research on neurocognitive aging has focused on the role of the cholinergic basal forebrain system, intermingled GABAergic cells are numerically as prominent and well positioned to regulate the activity of their cortical projection targets, including the hippocampus and prefrontal cortex. The effects of aging on noncholinergic BF neurons in primates, however, are largely unknown. In this study, we conducted quantitative morphometric analyses in brains from young adult (6 females, 2 males) and aged (11 females, 5 males) rhesus monkeys (*Macaca mulatta*) that displayed significant impairment on standard tests that require the prefrontal cortex and hippocampus. Cholinergic (ChAT^+^) and GABAergic (GAD67^+^) neurons were quantified through the full rostrocaudal extent of the BF. Total BF immunopositive neuron number (ChAT^+^ plus GAD67^+^) was significantly lower in aged monkeys compared with young, largely because of fewer GAD67^+^ cells. Additionally, GAD67^+^ neuron volume was greater selectively in aged monkeys without cognitive impairment compared with young monkeys. These findings indicate that the GABAergic component of the primate BF is disproportionally vulnerable to aging, implying a loss of inhibitory drive to cortical circuitry. Moreover, adaptive reorganization of the GABAergic circuitry may contribute to successful neurocognitive outcomes.

**SIGNIFICANCE STATEMENT** A long history of research has confirmed the role of the basal forebrain in cognitive aging. The majority of that work has focused on BF cholinergic neurons that innervate the cortical mantle. Codistributed BF GABAergic populations are also well positioned to influence cognitive function, yet little is known about this prominent neuronal population in the aged brain. In this unprecedented quantitative comparison of both cholinergic and GABAergic BF neurons in young and aged rhesus macaques, we found that neuron number is significantly reduced in the aged BF compared with young, and that this reduction is disproportionately because of a loss of GABAergic neurons. Together, our findings encourage a new perspective on the functional organization of the primate BF in neurocognitive aging.

## Introduction

Deficits in cognitive capacities mediated by the hippocampus and prefrontal cortex are well established consequences of aging (Burke and Barnes, 2006; Rapp et al., 2020). Age-related cognitive decline significantly impacts quality of life and, for many, results in loss of independence (Freedman et al., 2002). As people live longer, there is increased urgency to identify the neural substrates that mediate dysfunction, and to develop strategies to promote healthy cognitive aging.

The basal forebrain (BF), comprising the medial septum (MS), the vertical limb of the diagonal band of Broca (VDB), the horizontal limb of the diagonal band of Broca (HDB), the substantia innominata (SI), and the nucleus basalis of Meynert (nbM), innervates the hippocampus and entire neocortical mantle (Mesulam et al., 1983; Fisher et al., 1988; Freund and Antal, 1988; Gulyás et al., 1991; Gritti et al., 1997; Henny and Jones, 2008). Consistent with this extensive distribution of projections, the BF has been implicated in cortical activation, attention, and learning and memory (Olton et al., 1979; Walker and Olton, 1984; Givens and Olton, 1994; Voytko et al., 1994; Semba, 2000; Brown and McKenna, 2015). In humans, loss of cholinergic BF neurons is an early neurodegenerative hallmark of Alzheimer's disease (AD) and, consequently, this neuronal population has been studied extensively in the context of AD and related disorders (Whitehouse et al., 1982; Teipel et al., 2011; Grothe et al., 2012; Mesulam, 2012). Several studies report a decline of cholinergic BF integrity with normal aging (Fischer et al., 1989; Altavista et al., 1990; Armstrong et al., 1993; De Lacalle et al., 1996). The majority describe receptor and intracellular signaling alterations in association with disrupted cholinergic activity and cognitive decline, and that cholinergic neuron number remains relatively stable (Bartus et al., 1982; Mesulam et al., 1987; Lee et al., 1994; Davis et al., 1999; Gustilo et al., 1999; Fragkouli et al., 2005; Mufson et al., 2007; Ypsilanti et al., 2008; McQuail et al., 2011). Despite decades of research on the cholinergic BF in aging, evidence directly linking loss of these neurons to cognitive impairment is limited.

In addition to cholinergic neurons, the BF prominently includes a large population of codistributed inhibitory GABAergic neurons that, in species where it has been examined, also project to the hippocampus and neocortex (Fisher et al., 1988; Freund and Gulyás, 1991; Freund and Meskenaite, 1992; Gritti et al., 1993, 1997; McKenna et al., 2013). Numerically more prominent than the cholinergic component, GABAergic BF projection neurons influence hippocampal and cortical physiology as well as cognitive processes mediated by these brain regions (Freund and Antal, 1988; Burk and Sarter, 2001; Pang et al., 2001; Gritti et al., 2006; Kaur et al., 2008). An emerging literature suggests that, in rodents, alterations in inhibitory neuron groups, particularly BF GABAergic projections, may contribute to age-related cognitive decline (Rubio et al., 2012; Bañuelos et al., 2013; Spiegel et al., 2013). Cognitive aging across rodent models, nonhuman primates, and humans has also been linked to age-related shifts in excitatory/inhibitory (E/I) balance of relevant neural circuitry (Luebke et al., 2004; Bories et al., 2013; Thomé et al., 2016; Baker et al., 2019). Human imaging studies indicate that BF volume changes and gray matter alterations may precede both memory impairment and entorhinal cortex degeneration in the pathogenesis of AD (Schmitz and Nathan Spreng, 2016; Butler et al., 2018; Fernandez-Cabello et al., 2020). The assumption has been that changes in the cholinergic system underlie these results; the possibility that GABAergic BF alterations also contribute has received much less attention.

Here, we quantified BF cholinergic and GABAergic neuron number and volume in young and aged rhesus macaques behaviorally characterized using tasks critically dependent on medial temporal lobe and prefrontal cortex integrity (i.e., targets of projection neurons in the rostral and caudal BF, respectively; Mesulam et al., 1983; Pearson et al., 1983; Struble et al., 1986). The model represents a translational bridge to human aging, featuring similar neuroanatomy, age-related cognitive decline, and variable amyloid deposition, but without the widely distributed tauopathy and neuron loss characteristic of AD. This study aimed to document the anatomic integrity of these neuronal populations in the aged nonhuman primate brain, and to evaluate the results in relation to variability in cognitive outcome.

## Materials and Methods

### Subjects

Young adult (Y; age range, 7–13 years; mean age, 10.11 years; *n* = 8) and aged (age range at killing, 24–33 years; mean age at killing, 31.50 years; *n* = 16) rhesus monkeys (*Macaca mulatta*) of both sexes (young: 6 females, 2 males; aged: 11 females, 5 males) were used in this study. Monkeys were singly or pair housed and participated in long-term behavioral assessments at the California National Primate Research Center in Davis, California. Formal deprivation protocols were not used, but feeding was scheduled for after daily cognitive testing to motivate task performance. Water was available *ad libitum* in the home cage throughout. The vivarium was maintained on a 12 h light/dark cycle at an average ambient temperature of 23°C. Subjects were monitored for signs of stress or deteriorating health throughout the study. All experimental procedures were conducted in accordance with National Institutes of Health guidelines and following protocols approved by the Institutional Animal Care and Use Committees at the University of California.

### Behavioral characterization

Monkeys were tested as previously described on a standardized battery of learning and memory assessments including a prefrontal cortex-dependent delayed response (DR) task, and a delayed nonmatch-to-sample (DNMS) procedure that requires medial temporal lobe integrity (Rapp and Amaral, 1989, 1991; Rapp, 1990). Behavioral testing was conducted in a modified Wisconsin General Test Apparatus (WGTA; Harlow and Bromer, 1938). For each daily test session, subjects were transferred from the home cage to a sound-attenuating chamber where they had access to the WGTA three-well stimulus tray. Access to the stimulus tray was controlled by experimenter-operated screens (one opaque, the other transparent) that could be inserted between the caged monkey and the test tray. A one-way mirror allowed the experimenter to observe the performance of the subject undetected. A white noise generator masked ambient noise during training and testing.

#### Delayed response.

After animals adapted to displacing an object to retrieve food rewards in the WGTA, performance was assessed on a delayed response test of visuospatial working memory. The task consisted of an initial acquisition phase, followed by testing with successively longer delays, as described previously (Rapp et al., 2003). Briefly, trials were initiated when the opaque screen separating the monkey from the stimulus tray was raised. The transparent screen remained in place, allowing the monkey to observe while one of the lateral wells was baited with a food reward. Both lateral wells were then covered with identical plastic gray plastic plaques. During the acquisition phase, the transparent screen was raised as soon as the wells were covered (0 s delay) and subjects were allowed to displace one of the plaques, retrieving the reward if the correct location were selected. Subjects were provided 30 trials per day with a 20 s intertrial interval. The left and right wells were baited equally often in a pseudorandom sequence across trials. Acquisition training with a 0 s delay continued until monkeys reached a learning criterion of 90% correct (≤9 errors in 9 consecutive 10-trial blocks). Monkeys were then trained to the same criterion with a 1 s delay, imposed by lowering the opaque screen of the WGTA after baiting.

During subsequent testing, the memory demands of the task were progressively increased by introducing successively longer delays. Testing was the same as at the 1 s delay except that, after baiting, the opaque screen was lowered for a 5, 10, 15, 30, or 60 s retention interval. Testing continued for 30 trials a day for a total of 90 trials across three sessions at each delay interval.

#### Delayed non-matching-to-sample.

Next, monkeys were assessed on a DNMS task in which trials consisted of a sample object presentation followed by a recognition test. Trials began when the opaque screen was raised and the subject was presented with a novel sample stimulus (a “junk” object) covering the central, baited well of the WGTA. During the acquisition phase of the task, after subjects displaced the sample and obtained the food reward, the opaque screen was lowered for a 10 s delay. After the retention interval, the opaque screen was raised, and the sample was presented together with a novel object positioned over the lateral wells of the stimulus tray. Only the novel object was rewarded. A new pair of objects was presented on each trial. Monkeys were trained for 20 trials a day with a 10 s delay (intertrial interval, 30 s) until they reached a criterion of 90% correct across 100 consecutive trials. The left and right wells of the stimulus tray were baited equally often in each session. After the acquisition criterion was met, recognition memory demands were increased by imposing successively longer retention intervals of 15, 30, 60, and 120 s (100 trials total at each delay, 20 trials/d), and 600 s (50 trials total, 5 trials/d). Subjects remained in the test chamber for all retention intervals.

#### Histologic processing.

Following long-term behavioral testing, animals were deeply anesthetized and transcardially perfused with aldehyde fixatives. Perfusion began with a solution of cold 1% paraformaldehyde in 0.1 m phosphate buffer (PB), pH 7.4, for 2 min (250 ml/min) followed by 4% paraformaldehyde in 0.1 m PB for 1 h (10 min at 250 ml/min, followed by 50 min at 100 ml/min). The brains were blocked coronally, removed from the skull, and cryoprotected in a solution of 10% glycerol in PB containing 2% dimethyl sulfoxide (DMSO) for 1 d, followed successively by 20% glycerol in PB with and without DMSO for ∼3 d at 4°C. Brains were then rapidly frozen in isopentane chilled in a dry-ice ethanol bath, and stored at −80°C until histologic processing. Hemisectioned brains were sectioned at 40 µm on a freezing microtome in the coronal plane and stored as serial adjacent series in a cryoprotectant solution at −80°C.

#### Immunohistochemistry.

Two adjacent 1-in-10 series through the full rostrocaudal extent of the basal forebrain for each animal were processed immunocytochemically to detect cholinergic [choline acetyltransferase (ChAT)] and GABAergic [glutamic acid decarboxylase 67 (GAD67)] neurons. There is a wide diversity of inhibitory neuron subtypes in the mammalian brain, and, as a starting point toward establishing a comprehensive account, here GAD67 was selected as an especially widely used and well characterized marker for GABAergic cells. In addition, evidence in rats suggests that GAD67 preferentially stains projection neurons in the medial septum (Castañeda et al., 2005). Briefly, free-floating sections were washed three times in 0.1 m Tris-buffered saline (TBS; 100 mm Tris-HCl, 150 mm NaCl, pH 7.5) and incubated in 1% hydrogen peroxide solution for 30 min to quench endogenous peroxidases, followed by several TBS washes. The series processed for visualization of cholinergic neurons was preincubated in a blocking solution containing 1% bovine serum albumin (BSA), 10% normal horse serum (NHS), and 0.3% Triton X-100 in 0.1 m TBS for 1 h at room temperature and then incubated in primary antibody solution consisting of goat anti-ChAT (1:3000; catalog #AB144P, EMD Millipore), 1% BSA, and 10% NHS in 0.1 m TBS for 72 h at 4°C. The series processed for visualization of GABAergic neurons was first steamed in 10 mm EDTA for 5 min and allowed to return to room temperature before preincubation in a blocking solution composed of 1% BSA and 10% NHS in 0.1 m TBS for 1 h at room temperature. The series was then incubated in blocking solution that contained mouse anti-GAD67 (1:3000; catalog #MAB5406, EMD Millipore) at 4°C for 96 h. Following primary antibody incubation, the cholinergic and GABAergic series were washed three times in TBS and incubated in 0.1 m TBS containing 1% BSA and biotinylated horse anti-goat (1:1000; BA-9500, Vector Laboratories) and horse anti-mouse secondary antibody (1:2000; catalog #BA-2000, Vector Laboratories), respectively, for 2 h at room temperature. Finally, sections washed and incubated in avidin–biotin complex (diluted to half the concentration suggested by manufacturer; catalog #PK-6100, Vector Laboratories) for 1 h at room temperature. Immunopositive neurons were revealed using chromogen diaminobenzidine (catalog #SK-4100, Vector Laboratories; cholinergic reaction time, 6 min, GABAergic reaction time, 4 min). Sections were mounted onto gelatin-coated slides and allowed to dry at room temperature for 3–4 d, dehydrated in graded ethanol, cleared in xylene, and coverslipped with DPX Mounting Medium (catalog #13512, Electron Microscopy Sciences). A closely adjacent 1 in 10 series processed for visualization of Nissl bodies for each animal was used for anatomic reference.

It should be noted that, on the basis of the immunocytochemical approach used here, group differences in cell number could reflect either changes in the levels of the targeted markers or frank neuronal loss.

### Quantitative analysis

#### Delineation of the full extent of the basal forebrain.

**Figure 1.**
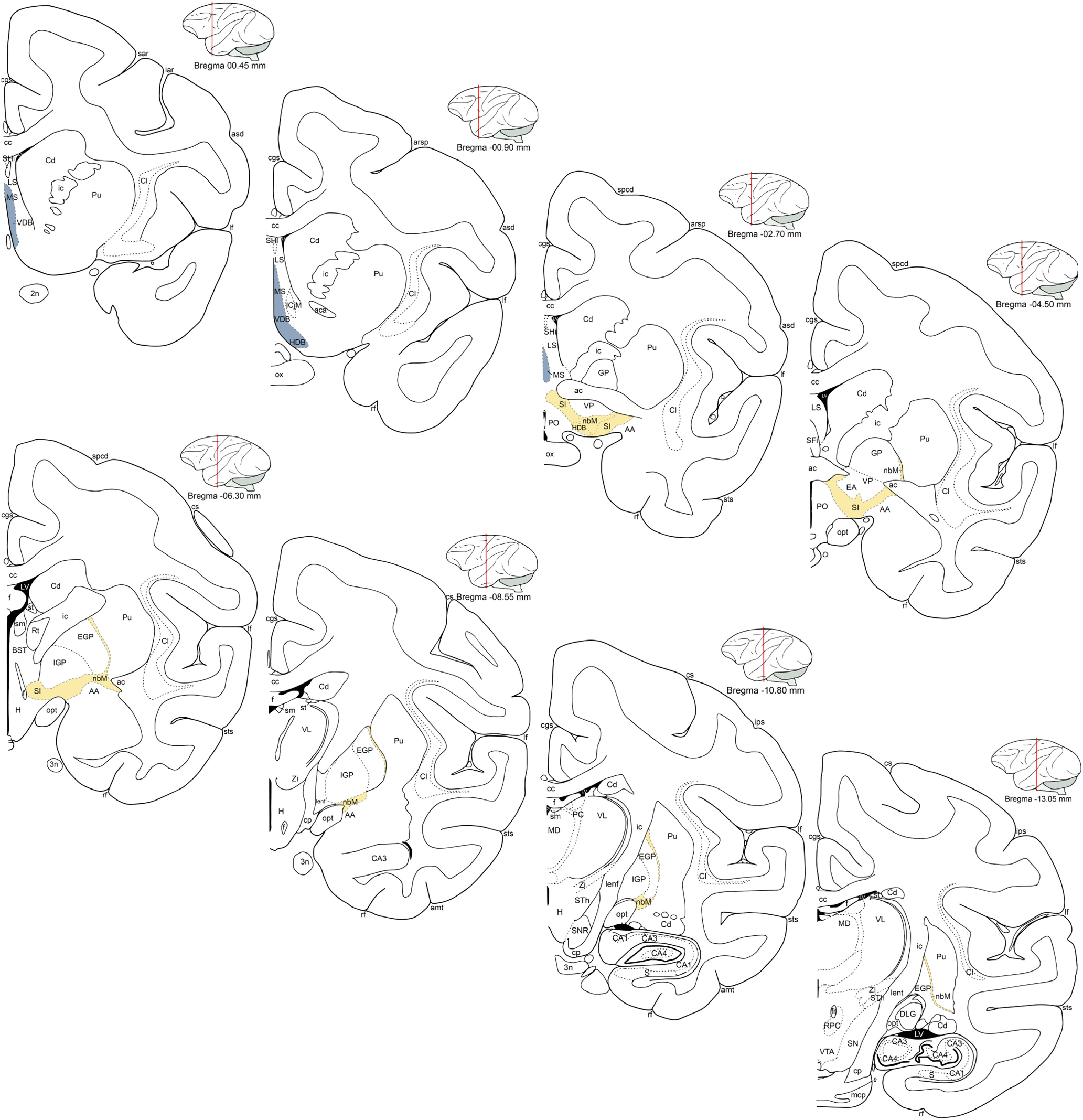
Basal forebrain regions included in morphometric estimates. Schematic illustrations modified from Paxinos et al. (2009) indicating the boundaries of the full extent of the basal forebrain (from Bregma 00.45 mm to Bregma −13.06 mm) and the delineation of rostral (blue) and caudal basal forebrain (yellow) examined in the current study. 2n, Optic nerve; AA, anterior amygdaloid area; aca, anterior commissure AcbC, nucleus accumbens core; amt, anterior middle temporal sulcus; arsp, arcuate sulcus spur; asd, anterior subcentral dimple; BST, bed nucleus of the stria terminalis CA1, field CA1 of hippocampus; CA3, field CA3 of hippocampus; CA4, field CA4 of hippocampus; cc, corpus callosum; Cd, caudate; cgs, cingulate sulcus; Cl, claustrum; cs, central sulcus; DLG, dorsal lateral geniculate nucleus; EA, extended amygdala; f, fornix; GP, globus pallidus; H, hypothalamus; iar, inferior arcuate sulcus; ic, internal capsule; If, lateral fissure; IGP, internal globus pallidus; ips, intraparietal sulcus; Lenf, lenticular fasciculus; LS, lateral septum; LV, lateral ventrical; mcp, middle cerebellar peduncle; MCPO, magnocellular preoptic area; MD, mediodorsal thalamic nucleus; opt, optic tract; ox, optic chiasm; PC, paracentral thalamic nucleus; PO, parieto-occipital area; Pu, putamen; rf, rhinal fissure; S, subiculum; sar, superior arcuate sulcus; SFi, septofimbrial nucleus; SHi, septohippocampal nucleus; sm, stria medullaris of the thalamus; SN, substantia nigra; spcd, superior precentral dimple; st, stria terminalis; STh, subthalamic nucleus; sts, superior temporal sulcus; Tu, olfactory tubercle; VP, ventral pallidum; VTA, ventral tegmental area; ZI, zona incerta.

Basal forebrain borders were first delineated in the ChAT immunopositive series under low-power magnification (1.6× objective) using a light microscope equipped with a CCD camera and a motorized stage, interfaced with a quantitative morphometry system (MBF BioScience). Digitized borders generated using the ChAT immunopositive series were then overlaid on images of adjacent sections processed for visualization of GAD67^+^ cells. Contours were adjusted as needed to accommodate slight anatomic and orientation differences in the adjacent sections. The region of interest was defined as the location of ChAT immunopositive cells throughout their rostrocaudal extent in the BF. Rostrally, the Ch1 cells of the medial septal nucleus emerged in sections after the rostrum of the corpus callosum was no longer visible and continued to the vertical and horizontal limbs of the diagonal band of Broca where the Ch2 and then Ch3 groups appeared, respectively. The Ch4 was the most posterior cell group to emerge and basal forebrain contours continued through the caudal extent of the nucleus basalis of Meynert (Mesulam et al., 1983, 1984), where ChAT immunopositive cells were no longer discernable. This parcellation yielded ∼37 slices per brain for quantification (mean, 36.88, SD, 4.89; spacing, 400 µm). To assess potential regional effects, Ch1 and Ch2 cells, which include neurons that disproportionately project to the hippocampus, were grouped as the rostral division of the BF for some analyses, comprising neurons in the MS, VDB, and the anterior portion of the HDB. Ch3 and Ch4 neurons, which originate projections predominately to the neocortex, were designated as the caudal division of the BF, including cells in the posterior HDB, SI, and nbM (Fig. 1).

#### Estimation of ChAT immunopositive neuron number and size.

A pilot study using a full ChAT series from a representative brain revealed that the stereological sampling necessary to achieve adequate precision in the population estimate of total cell number would not be more efficient than exhaustive counting. This was largely because of the highly variable packing density of cholinergic cells in the basal forebrain. Accordingly, ChAT immunopositive neurons were exhaustively counted using an LED light microscope (model DM4000 B, Leica) equipped with a CCD camera (model C11440, Hamamatsu) and outfitted with a motorized stage (model H101F Prior Scientific) controlled with StereoInvestigator software (version 2018 1.1; MBF BioScience). The stage was moved between contiguous fields so that all cells within the region of interest were counted at high-power magnification using a 40× objective. The total number of cholinergic neurons in one hemisphere of the basal forebrain was estimated by multiplying the number of cells counted by 10 (i.e., the reciprocal of the fraction of serial sections analyzed).

Neuronal soma volume was estimated using the nucleator probe in parallel with cell number quantification (Dorph-Petersen et al., 2004; Lemus et al., 2015). Briefly, for every 20th cell counted, the nucleator probe function in StereoInvestigator generated randomly oriented orthogonal lines radiating from the center of the cell where the marker was placed. The intersection of the lines with the cell body wall was marked and total cell volume was calculated by taking the third power of the measurements. In its strictest form, the nucleator method is implemented in uniformly random or vertical uniformly random sections, to ensure unbiased sampling. Such preparations are impractical in most neuroanatomical applications, however, and they are incompatible with standard pipelines for cell number quantification. Although the standard coronal sections analyzed in the current study could, in principle, introduce measurement bias for cells with nonrandom orientation, there is no reason to suspect a disproportionate influence on the size estimates for any of the experimental groups examined. The CE values of <0.01 demonstrated robust sampling precision (Table 1).

**Table 1.** Nucleator probes to quantify size of ChAT and GAD67 immunopositive cells in basal forebrain of young and aged behaviorally characterized monkeys

Object	Average objects measured ± SD	Average CE	CV
ChAT^+^ cells	763 ± 130.03	0.005	0.25
Rostral ChAT^+^ cells	205 ± 61.30	0.012	0.44
Caudal ChAT^+^ cells	558 ± 100.23	0.007	0.53
GAD67^+^ cells	350 ± 142.63	0.003	0.39
Rostral GAD67^+^ cells	140 ± 64.18	0.007	0.63
Caudal GAD67^+^ cells	210 ± 98.45	0.005	0.44

CV, Coefficient of variation.

#### Estimation of GAD67 immunopositive neuron number and size.

The immunohistochemical preparations from two monkeys (one young and one aged) were not suitable for quantification because of uneven, blotchy staining, yielding a final *N* of 22 (young, *n* = 7; aged, *n* = 15) for assessing GABAergic neuron number and size in the basal forebrain.

GAD67 immunopositive cell number was quantified using the optical fractionator, an unbiased, design-based stereological method (West et al., 1991). Starting at a randomly selected level within the first sampling interval, quantification was performed using a DMRB light microscope (Leica), equipped with a CCD camera and a motorized stage (model Mac 6000, Ludl Electronic Products) controlled by StereoInvestigator software (version 2018 1.1; MBF BioScience). The motorized stage of the microscope was moved in evenly spaced x–y intervals under computer control, surveying the regions of interest in each section according to a systemic random sampling scheme (Table 2, sampling details). Section thickness was measured at each sampling site using a 60× oil-immersion objective (numerical aperture, 1.4). Quantification was confined to an optical disector 14 µm in height, positioned 1.5 µm below the cut surface of the histologic section. An immunopositive cell body was only counted when it first came into focus within the optical disector and did not touch the exclusion lines of the counting frame (Sterio, 1984; Gundersen, 1986). The total number of GAD67 immunopositive cells in the basal forebrain was estimated as the product of the cells counted in a known, uniformly random sample of the region of interest, multiplied by the reciprocals of the sampling fraction, the x–y area, and the tissue thickness sampling fractions. The tissue thickness sampling fraction was calculated as the number-weighted mean section thickness, which accounts for tissue shrinkage in the *z*-axis (Dorph-Petersen et al., 2001). Stereological sampling parameters are listed in Table 2.

**Table 2.** Sampling parameters used for estimating total number of GABAergic basal forebrain neurons

Object	Sampling grid (µm)	Counting frame (µm)	Disector height (µm)	Average no. of sections	Average object counted ± SD	Average CE*^a^*	CV
GAD67^+^ cells	400 × 400	70 × 70	14	37	350 ± 143	0.06	0.19
Rostral GAD67^+^ cells	400 × 400	70 × 70	14	13	140 ± 64	0.09	0.23
Caudal GAD67^+^ cells	400 × 400	70 × 70	14	24	210 ± 98	0.07	0.23

Total cell numbers were estimated using the following formula: *N* (total number) = 1/section sampling fraction (ssf) × 1/area sampling fraction (asf) × 1/height sampling fraction (hsf) × number of immunopositive cells counted. The total number of cells counted for each subject and the sampling grid and counting frame used to generate the asf for each label are provided above. The ssf in the current study equaled one-tenth. The hsf was calculated using the mean weighted thickness (with section thickness measured at each sampling site). CV, Coefficient of variation.

*^a^*Gundersen's CE, *m* = 1.

GABAergic neuronal soma volume was quantified using the nucleator probe during optical fractionator cell number quantification. For each GAD67 immunopositive cell counted, cell volume was also measured as described above.

Coefficients of error (CEs) were calculated to determine the precision of the neuron count population estimates and soma volumes. Equations used to generate the population estimate CEs are based on Gundersen's smoothness classification m = 1, as the areas defined for the cell counts changed smoothly from the rostral emergence of the MS to the caudal extent of the nucleus basalis of Meynert (Gundersen et al., 1999). The CEs (range, 0.06–0.09) were less than half the observed variation across subjects (coefficient of variation range, 0.19–0.23; Table 2), indicating that the sampling and counting parameters were sufficiently precise to detect true biologically driven differences in estimated total neuron number among experimental groups (Gundersen and Jensen, 1987; West, 1999; Dorph-Petersen et al., 2001; Boyce et al., 2010).

### Statistical analysis

#### Behavioral assessment.

Comparisons between age groups for task acquisition were conducted using independent two-tailed *t* tests. Performance during the delay phase of the DR and DNMS procedures was compared using two-factor repeated-measures ANOVA (age × delay). To directly assess the relationship between memory and cell number or volume, the mean percentage correct averaged across postacquisition delays (DR: 5, 10, 15, 30, and 60 s; DNMS: 15, 30, 60, 120, and 600 s) was calculated and used as a summary performance score.

#### Cell count and size comparisons.

Estimates of cholinergic (ChAT^+^), GABAergic (GAD67^+^), and total (ChAT^+^ plus GAD67^+^) basal forebrain neuron number and volumes were compared between age and cognitive group using independent sample *t* tests and one-factor ANOVAs with Tukey's *post hoc* analyses where appropriate. Relationships between cell number and volume with performance on the DR and DNMS tasks were tested using Pearson's correlations. To reach sufficient sample size, and young and aged data were collapsed. Descriptive data are presented as the mean ± SEM. All statistical analyses were conducted using SPSS Statistics 28 and GraphPad Prism 9. *p*-Values < 0.05 were considered significant.

#### Sex differences.

As reported in previous studies (Bachevalier and Hagger, 1991; Rapp et al., 2003; Baxter et al., 2023), no differences were observed in DR and DNMS performance between male and female monkeys (DR: repeated-measures ANOVA, main effect of sex: young: *F*_(1,6)_ = 0.325, *p* = 0.573; aged: *F*_(1,14)_ = 0.693, *p* = 0.408; DNMS: repeated-measures ANOVA, main effect of sex: young: *F*_(1,6)_ = 0, *p* = 0.991; aged: *F*_(1,14)_ = 0.674, *p* = 0.415), and, consequently, sex differences were not a focus of the current analysis. In addition, nearly twice as many females as males were examined (young: 2 males, 6 females; aged: 5 males, 11 females). Future studies with sufficient numbers of animals of both sexes are needed to determine whether any sex differences in the morphometric data are reliable and vary as a function of age.

## Results

### Aged rhesus monkeys display cognitive impairment and substantial individual variability in performance

**Figure 2.**
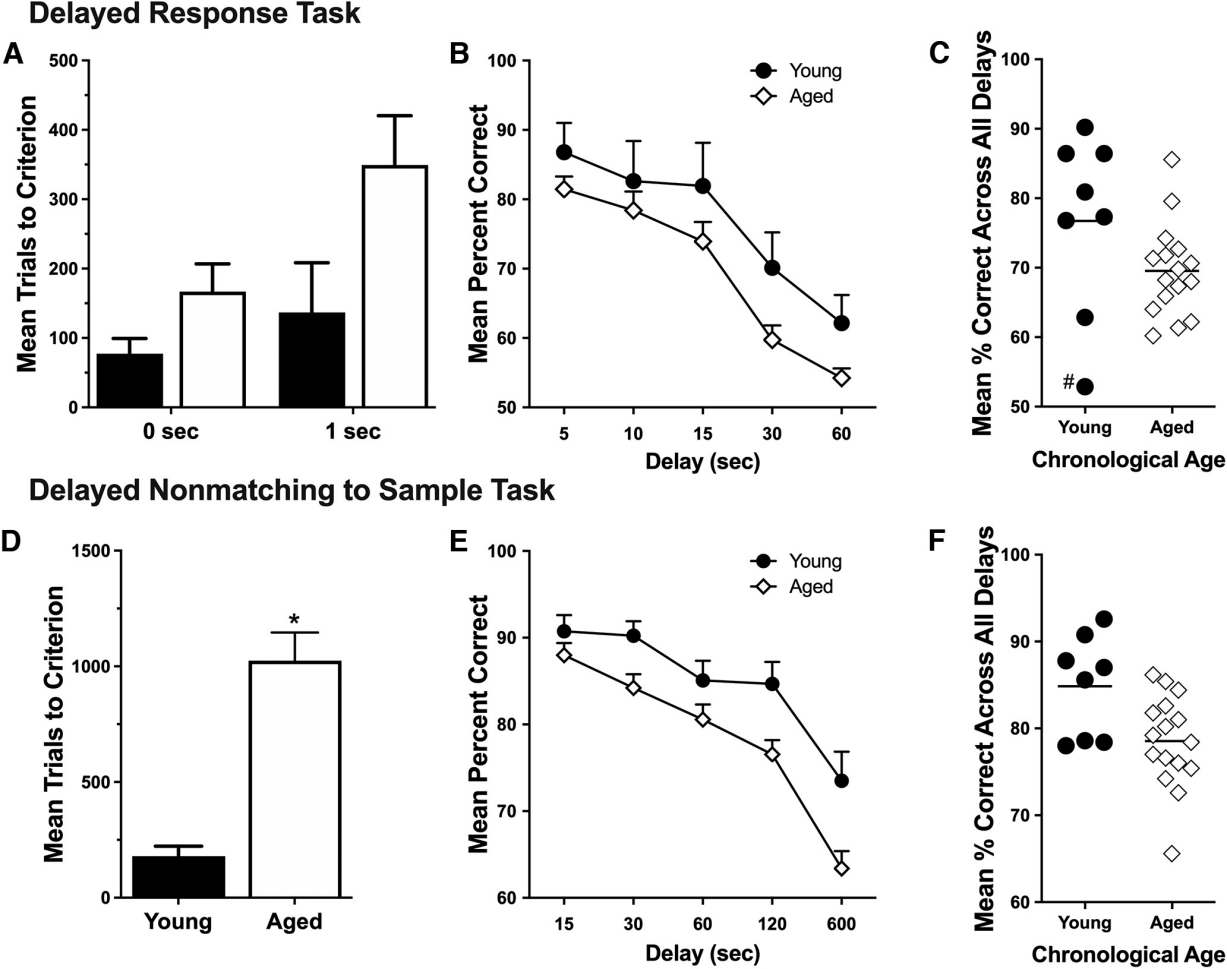
Cognitive performance in young and aged monkeys. ***A***, Mean number of trials to reach the acquisition criterion (±SE) on DR task with 0 and 1 s delays (young monkeys: filled bars, *n* = 8; aged monkeys: open bars, *n* = 16). ***B***, Mean percentage correct across increasing retention intervals (±SE) on the DR task in young and aged monkeys. ***C***, Scatter plots of scores for individual young and aged monkeys plotted as the average percentage correct across all testing trial delays. # identifies one young monkey that performed worse on this task than all other monkeys. ***D***, Aged monkeys required significantly more trials to reach criterion in the training phase of the delayed nonmatching-to-sample task. ***E***, In the testing phase, aged monkeys perform worse than young monkeys. ***F***, DR task performance of individual monkeys plotted by the mean percentage correct across all delays. *p < 0.05.

Performance of the young and aged monkeys on the delayed response and delayed nonmatching to sample tasks conformed to many previous descriptions (Moss et al., 1988; Rapp and Amaral, 1989; Bachevalier et al., 1991; Herndon et al., 1997; Fletcher and Rapp, 2012; Morrison and Baxter, 2012; Gray and Barnes, 2019; Cooper et al., 2022). Briefly, young and aged monkeys learned the DR task at the 0 delay at similar rates (unpaired *t* test: *t*_(22)_ = 1.524, *p* = 0.142), demonstrating that both groups were motivated to perform the task in the absence of a significant memory load. At the 1 s delay, aged monkeys required more than twice as many trials to reach the acquisition criteria as young monkeys (Fig. 2*A*). This difference was not statistically significant, however, because of the outlying score of a single young animal that required more trials than any other young monkey in the experiment to reach criteria (unpaired *t* test; all monkeys: *t*_(22)_ = 1.882, *p* = 0.073; excluding young outlier: *t*_(21)_ = 3.720, *p* = 0.002).

During DR testing with delays, accuracy in both young and aged monkeys declined as the retention interval increased and information had to be held in working memory for longer periods (repeated-measures ANOVA; main effect of delay: *F*_(4,88)_ = 53.76, *p* < 0.001; Fig. 2*B*). The aged monkeys as a group scored worse than young. The same young monkey that performed poorly during acquisition also scored below any other animal on DR delays, averaging just above chance (52.88%) across the 5–60 s intervals (Fig. 2*C*, identification). The difference in performance between age groups was statistically significant only when values for this outlier were excluded (with animal included, repeated-measures ANOVA, main group effect: *F*_(1,22)_ = 3.341, *p* = 0.081; with animal excluded, main group effect: *F*_(1,21)_ = 9.887, *p* = 0.005). Otherwise, behavioral and morphometric data for this monkey were similar to other young animals and were therefore included in the analysis.

On the DNMS task, aged monkeys as a group required many more trials than young to reach the training criteria with a 10 s delay (unpaired *t* test: *t*_(22)_ = 4.793, *p* < 0.001; Fig. 2*D*). While recognition accuracy declined as the delay interval increased for both young and aged monkeys (repeated-measures ANOVA, main effect of delay: *F*_(4,88)_ = 71.86, *p* < 0.001), the aged group performed significantly worse than young (repeated-measures ANOVA, main effect of group: *F*_(1,22)_ = 7.122, *p* = 0.014). There was a trend level delay by age interaction (*F*_(4,88)_ = 2.271, *p* = 0.068) and planned comparisons confirmed that age group differences were statistically significant selectively at the longest delays (unpaired *t* tests; 120 s delay: *t*_(22)_ = 2.796, *p* = 0.011; 600 s delay: *t*_(22)_ = 2.746, *p* = 0.012) when visual recognition memory load was greatest (Fig. 2*E*).

To relate the neuroanatomical findings to the cognitive data, summary scores of DR and DNMS task accuracy were calculated for individual animals as their mean percentage correct across all postacquisition retention delays (DR, 5–60 s; DNMS, 15–600 s). As shown in Figure 2, *C* and *F*, there was tremendous individual variability in both delayed response and visual recognition memory performance among the aged monkeys. Aged animals that performed within the range of young were classified as aged unimpaired (AU; DR: mean percentage correct, ≥70% excluding two outlying young values; DNMS: mean percentage correct, ≥78%), and those that performed worse than young (DR: mean percentage correct, <70% excluding two outlying young values; DNMS: mean percentage correct, <78%) were classified aged impaired (AI).

The monkeys examined in this analysis participated in a larger, long-standing research program on neurocognitive aging in nonhuman primates (Shamy et al., 2006; Dumitriu et al., 2010; Long et al., 2020; Cooper et al., 2022). Notably, the behavioral data for this subsample of subjects are fully consistent with results from the larger population from which they were derived (Baxter et al., 2023). Similar to the population data reported by Baxter et al. (2023), there was no significant relationship between performance on the DNMS and DR tasks among the aged monkeys in the current study (Pearson's *r* = 0.056, *p* = 0.7963, *n* = 16). These findings align with a long history of neuropsychological research (Rapp and Amaral, 1989, 1991; Comrie et al., 2018) suggesting that DR and DNMS measure distinct cognitive capacities and justifying considering them separately in relation to the morphometric results. Interestingly, as also reported in Baxter et al. (2023), among the aged monkeys alone, there was no significant relationship between chronological age at testing and DNMS or DR performance (DR: Pearson's *r* = 0.43, *p* = 0.0931, *n* = 16; DNMS: Pearson's *r* = 0.10, *p* = 0.6999, *n* = 16).

### Cholinergic and GABAergic neuronal populations are codistributed across the full extent of the basal forebrain

**Figure 3.**
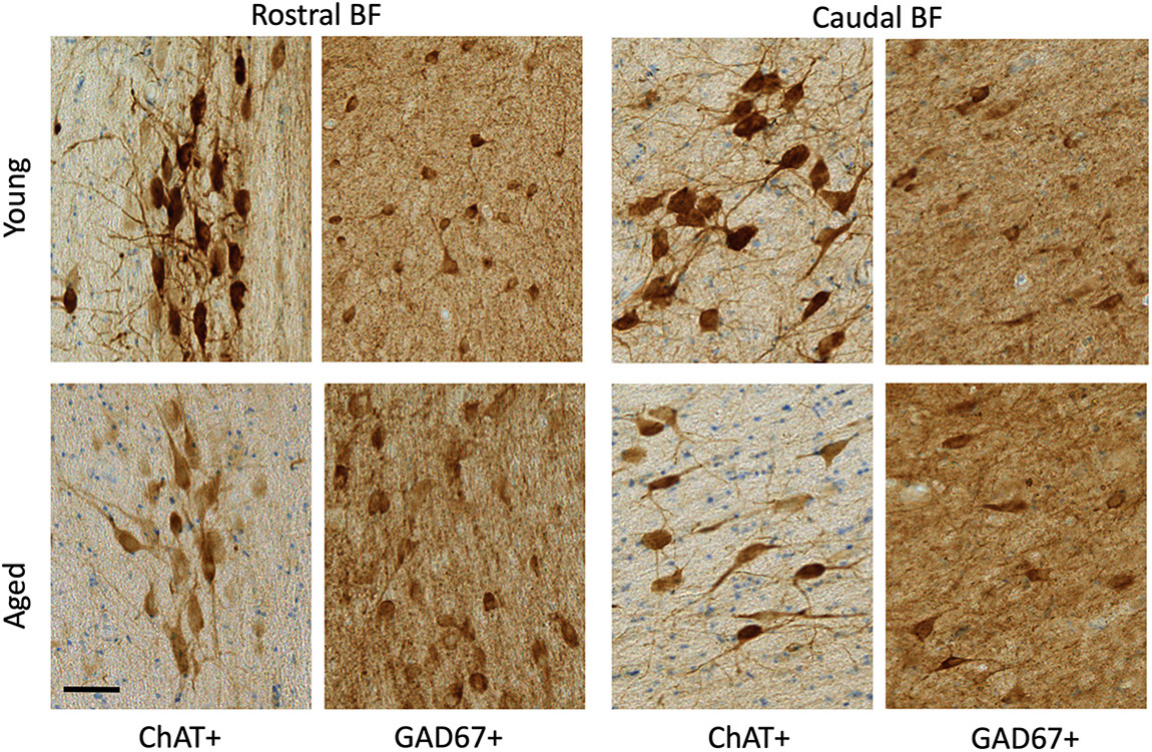
Representative photomicrographs of ChAT (with Nissl counterstain) and GAD67 immunopositive cells in the MS (rostral, Bregma = 0.45 mm) and nbM (caudal −6.30) nuclei of basal forebrains of young and aged monkeys. Bregma locations are illustrated in Figure 1. Scale bar, 50 µm.

ChAT immunopositive cells were clustered throughout ∼14 mm along the full rostral–caudal extent of the basal forebrain, spanning rostrally from the MS and continuing through the caudal portion of the nbM (Fig. 1). In both age groups, GABAergic neuronal populations were coextensive and partially intermingled with cholinergic neurons throughout the basal forebrain (Fig. 3). While cholinergic neurons tended to be clustered, GABAergic neurons were more homogeneously distributed throughout the region, but exhibited diverse size and morphologies including multipolar, fusiform, and oval cell bodies. Consistent with previous qualitative observations (Sarter and Bruno, 2002; Gritti et al., 2006), GABAergic basal forebrain neurons were significantly more numerous than cholinergic neurons (unpaired *t* test: *t*_(44)_ = −1.554, *p* = 0.005; Table 3). Independent of age, GABAergic basal forebrain neurons were significantly smaller than cholinergic neurons (unpaired *t* test: *t*_(44)_ =15.279, *p* < 0.001; Table 3). In both cholinergic and GABAergic populations, cells in the rostral division were significantly smaller than magnocellular neurons in the caudal division (ChAT rostral vs caudal unpaired *t* test: *t*_(46)_ = −3.598, *p* < 0.001; GAD67 rostral division vs caudal division: *t*_(42)_ = −3.330, *p* = 0.002; Table 3). Consistent with previously reported data from rats and cats (Brashear et al., 1986; Takeuchi et al., 2021), the cell volume estimates reported here indicate that the size distributions of ChAT and GAD67 immunopositive neurons in the monkey BF are essentially nonoverlapping.

**Table 3.** Cholinergic and GABAergic basal forebrain neuron number and size in all (young and aged) monkeys

	Cell (*n*)		Cell volume (µm^3^)	
	ChAT^+^	GAD67^+^	ChAT^+^	GAD67^†^
BF	148,265	168,501*	7014	2225*
(SE; *n*)	(4951; 24)	(12,494; 22)	(288; 24)	(92; 22)
Rostral	36891	11,582*	5840†	1957*^†^
(SE; *n*)	(2364; 24)	(5828; 22)	(341; 24)	(104; 22)
Caudal	111,295	101,245	7460	2411*
(SE; *n*)	(3713.42; 24)	(9297; 22)	(294; 24)	(89; 22)

*p < 0.05 relative to ChAT.

^†^*p* < 0.05 relative to caudal.

### Total basal forebrain neuron number (ChAT^+^ plus GAD67^+^) is significantly reduced in the aged monkey

**Figure 4.**
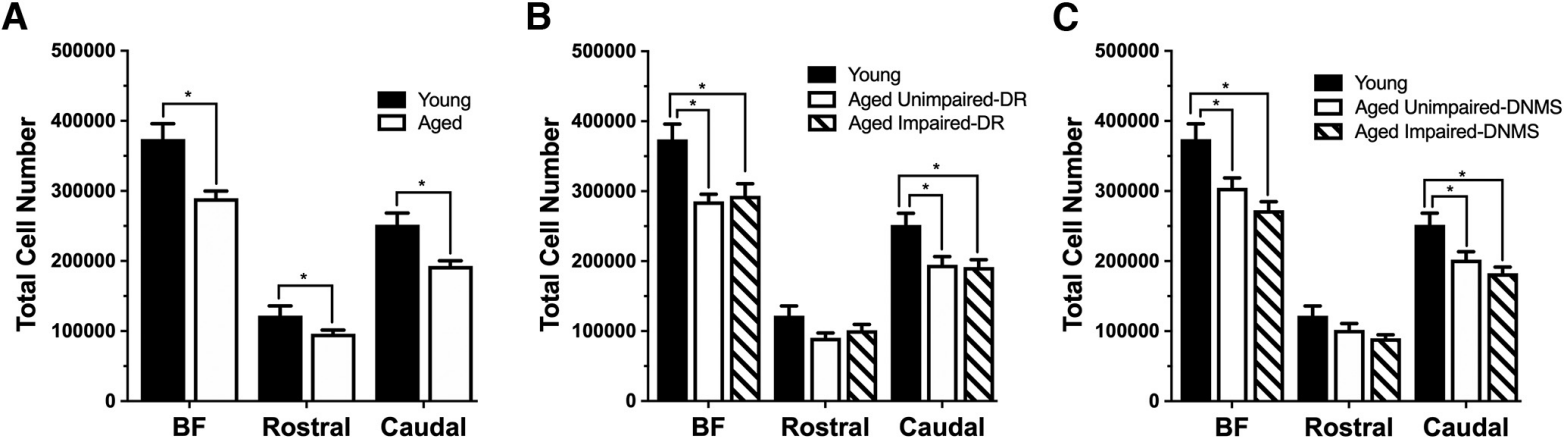
Mean estimated total immunopositive neuron number (ChAT plus GAD67; ±SE) in young and aged behaviorally characterized monkeys. ***A***, Total cell number (ChAT+ plus GAD67+) in the full extent of the basal forebrain, and in the rostral and caudal divisions were significantly reduced in aged monkeys (*n* = 16) compared with young monkeys (*n* = 8). ***B***, Total neurons were significantly reduced in the entire basal forebrain and the caudal nuclei of aged monkeys characterized as impaired and unimpaired on the delayed response (DR) task. ***C***, Similar reductions were observed in young and aged animals characterized by their performance in the nonmatch to sample task (DNMS). *p < 0.05.

Total basal forebrain neuron number was estimated by summing the counts for ChAT and GAD67 immunopositive cells. Labeled cells were significantly fewer in number across the full extent of the basal forebrain in aged monkeys compared with young (unpaired *t* test: *t*_(20)_ = 4.059, *p* < 0.001); this difference was observed in both the rostral division (unpaired *t* test: *t*_(20)_ = 2.115, *p* = 0.047) and caudal division (unpaired *t* test: *t*_(20)_ = 3.763, *p* = 0.001; Fig. 4*A*). A comparison of total cell number (ChAT^+^ plus GAD67^+^) across behavioral subgroups characterized on both the DR and DNMS tasks revealed significant differences in the full extent of the basal forebrain (DR task main group effect: *F*_(2,19)_ = 7.935, *p* = 0.003; DNMS task main group effect: *F*_(2,19)_ = 9.591, *p* = 0.001) and the caudal BF (DR task main group effect: *F*_(2,19)_ = 6.757, *p* = 0.006; DNMS task main group effect: *F*_(2,19)_ = 7.769, *p* = 0.003). However, pair-wise contrasts failed to reveal a significant difference between aged subgroups classified as impaired and unimpaired (Fig. 4*B*,*C*).

**Figure 5.**
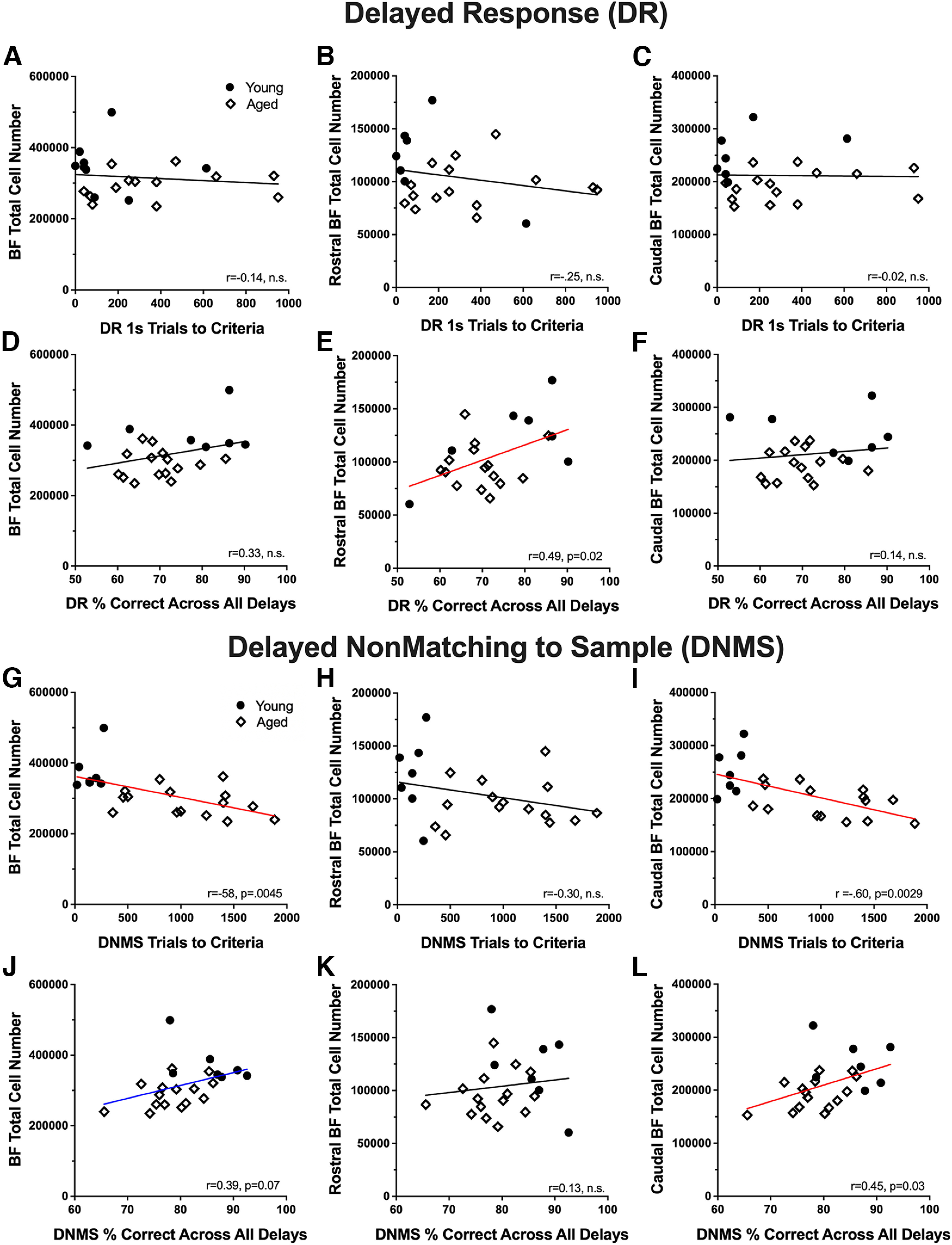
Relationship between total basal forebrain cell number (ChAT^+^ plus GAD67^+^) and performance on behavioral tasks. ***A–C***, Correlations between total cell numbers in the full extent of the basal forebrain (***A***), the rostral BF (***B***), the caudal BF (***C***), and the number of trials needed to reach criterion after a 1 s delay during the acquisition phase of the delayed response task (DR). ***D–F***, Scatter plots illustrating the relationship between total cell numbers in the full extent of the basal forebrain (***D***), the rostral BF (***E***), the caudal BF (***F***), and the average percentage of correct choices across all delays during the testing phase of the DR task. ***G–I***, Correlations between total cell numbers in the full extent of the basal forebrain (***G***), the rostral BF (***H***), the caudal BF (***I***), and the number of trials needed to reach criteria during the acquisition phase of the nonmatch to sample task (DNMS). ***J–L***, Relationships between total cell numbers in the full extent of the basal forebrain (***J***), the rostral BF (***K***), the caudal BF (***L***), and the mean percentage of correct choices across all delays during the testing portion of the DNMS task. Red regression lines represent significant relationships, *p* < 0.05. Blue regression line represents 0.05 > *p* < 0.10.

The relationship between total BF cell number and cognitive performance was further explored using Pearson's *r* correlations. Table 4 summarizes the results. For both the DR and DNMS tasks, the number of trials needed to reach criterion during the acquisition phase, and the average percentage correct across memory delays, were used as summary performance metrics. In the DR procedure, as shown in Figure 5*A–C*, total basal forebrain cell number was not significantly associated with task acquisition. There was, however, a significant positive correlation between the number of immunopositive cells in the rostral division and DR delay performance such that monkeys with higher numbers scored more accurately (*r* = 0.49, *p* = 0.02; Fig. 5*E*). For DNMS, cell number in the full extent of the BF and the caudal division were both significantly and negatively associated with the number of trials needed to reach the acquisition criterion, such that monkeys with lower cell numbers were slower to learn the procedure [BF: *r* = –0.58, *p* = 0.005 (Fig. 5*G*); caudal BF: *r* = –0.60, *p* = 0.003 (Fig. 5*I*)]. Cell number in the caudal division also correlated significantly with delay performance on DNMS; monkeys with more cells displayed better visual object recognition memory (*r* = 0.45, *p* = 0.03; Fig. 5*L*). No statistically significant relationship was found between chronological age and basal forebrain cell numbers when young and aged monkeys were considered separately.

**Table 4.** Pearson correlation coefficients and significance levels for basal forebrain neuronal populations in young and aged monkeys

	DR TTC		DR % Accuracy		DNMS TTC		DNMS % Accuracy	
	*r*	*p*-value	*r*	*p*-value	*r*	*p*-value	*r*	*p*-value
Cell Number								
Total BF cells	−0.14	0.547	0.33	0.129	**−0.58**	**0.005**	0.39	0.071
Total rostral cells	−0.25	0.265	**0.48**	**0.021**	−0.30	0.176	0.13	0.560
Total caudal cells	−0.02	0.919	0.14	0.527	**−0.60**	**0.003**	**0.45**	**0.034**
ChAT^+^ BF cells	−0.31	0.142	**0.47**	**0.020**	0.08	0.697	0.07	0.747
ChAT^+^ rostral cells	0.01	0.962	0.18	0.392	0.35	0.089	−0.26	0.226
ChAT^+^ caudal cells	**−0.42**	**0.039**	**0.51**	**0.010**	−0.11	0.594	0.26	0.218
GAD67^+^ BF cells	−0.04	0.865	0.17	0.438	**−0.64**	**0.001**	0.35	0.113
GAD67^+^ rostral cells	−0.29	0.194	**0.46**	**0.033**	**−0.47**	**0.028**	0.24	0.276
GAD67^+^ caudal cells	0.13	0.566	−0.05	0.841	**−0.57**	**0.006**	0.32	0.153
Volume								
ChAT^+^ BF cells	0.24	0.251	−0.254	0.230	0.04	0.866	0.05	0.827
ChAT^+^ rostral cells	0.09	0.663	−0.20	0.360	−0.002	0.992	0.15	0.492
ChAT^+^ caudal cells	0.28	0.186	−0.26	0.212	0.07	0.761	−0.02	0.917
GAD67^+^ BF cells	−0.49	0.909	0.15	0.728	0.37	0.361	−0.51	0.193
GAD67^+^ rostral cells	0.19	0.649	0.19	0.640	0.34	0.406	−0.08	0.856
GAD67^+^ caudal cells	−0.25	0.545	0.21	0.618	0.37	0.373	−0.65	0.084

Total, ChAT^+^ plus GAD67^+^; TTC, trials to criterion. Bold type indicates *p* < 0.05.

### Cholinergic neuron number remains stable in the rostral BF but is reduced caudally in aging

**Figure 6.**
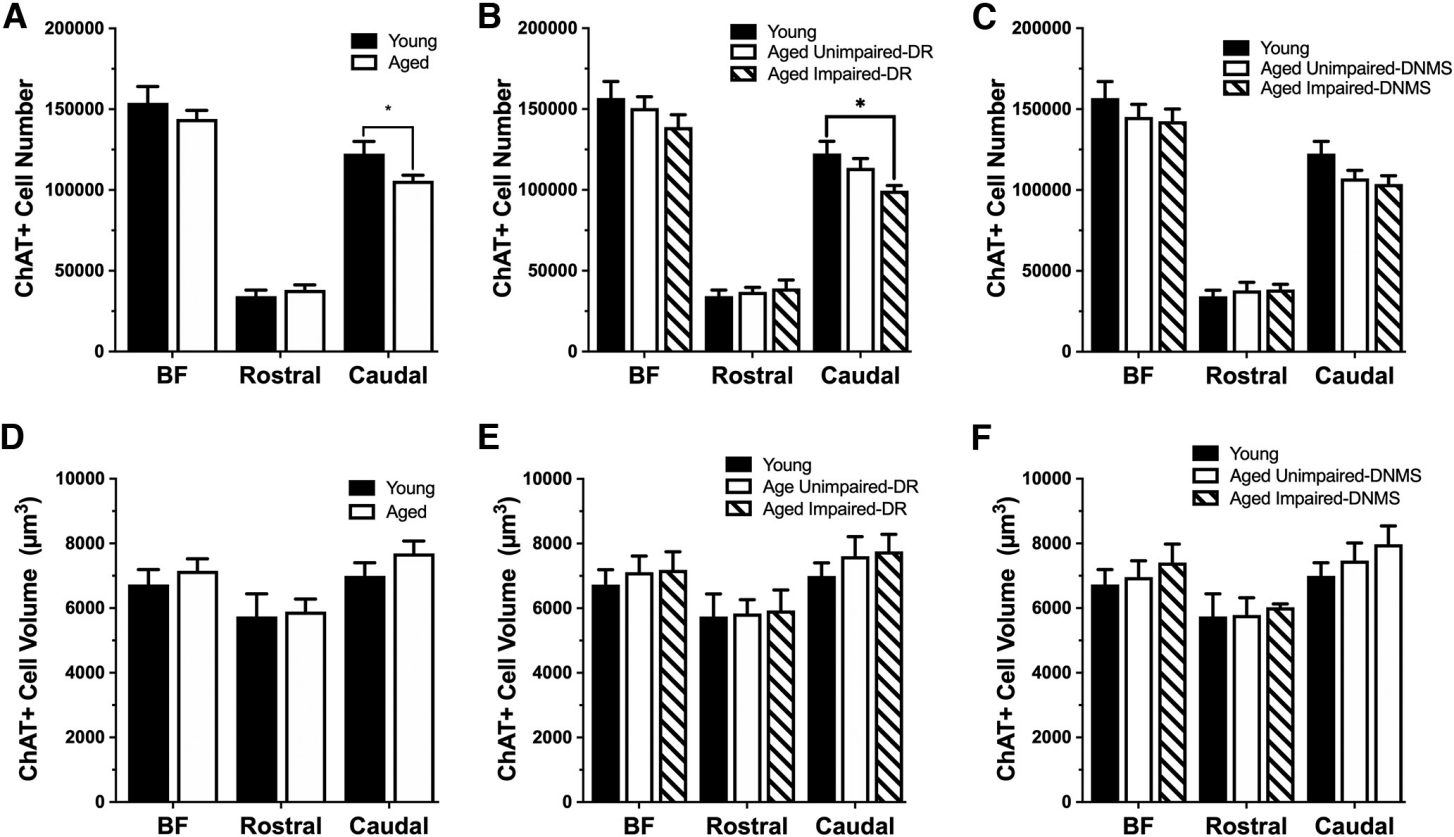
Mean (±SE) cholinergic (ChAT^+^) cell number and volume in the entire basal forebrain, rostral division, and the caudal division in young (*n* = 8) and aged (*n* = 16) behaviorally characterized monkeys. ***A***, Cholinergic cell number was significantly lower only in the caudal aged BF, not in the full extent of the BF or rostral BF. ***B***, ***C***, This significant difference was also observed in the aged subgroup characterized as impaired on the delayed response task (***B***), but no difference was found when grouped by delayed nonmatch-to-sample performance (***C***). ***D–F***, There was no difference in cholinergic cell volume between young and aged monkeys or among the aged behavioral subgroups. *p < 0.05.

Basal forebrain ChAT^+^ cell numbers in the full and rostral extent were comparable across groups but selectively decreased with aging in the caudal division. Specifically, the cholinergic cell number across the full extent of the basal forebrain did not differ between young and aged monkeys (unpaired *t* test: *t*_(22)_ = 1.245, *p* = 0.226; Fig. 6*A*) or between young, aged unimpaired, and aged impaired monkeys [one-way ANOVA; DR task main group effects: *F*_(2,21)_ = 1.247, *p* = 0.308 (Fig. 6*B*); DNMS task: *F*_(2,21)_ = 0.764, *p* = 0.478 (Fig. 6*C*)]. This was also true of cholinergic populations in the rostral division [age effect, unpaired *t* test: *t*_(22)_ −0.759, *p* = 0.456 (Fig. 6*A*); Y, AU, AI group effect, one-way ANOVA: DR task: *F*_(2,21)_ = 0.338, *p* = 0.717 (Fig. 6*B*); DNMS task: *F*_(2,21)_ = 0.278, *p* = 0.760 (Fig. 6*C*)]. However, cholinergic neuronal populations in the caudal division, known to project to the prefrontal cortex (Mesulam et al., 1983), were significantly decreased in aged monkeys compared to young monkeys (unpaired *t* test: *t*_(22)_ = 2.338, *p* = 0.029; Fig. 6*A*, Table 5). While there was a numerical decrease in caudal cholinergic populations in the basal forebrain of monkeys characterized as AU and AI on the DNMS task compared with young (one-way ANOVA; *F*_(2,21)_ = 2.707, *p* = 0.090; Fig. 6*C*), the group difference only reached statistical significance when the aged monkeys were classified as AU or AI based on their performance on the DR task (one-way ANOVA; *F*_(2,21)_ = 4.529, *p* = 0.023; Fig. 6*B*). As observed for the combined population above, no significant difference in ChAT^+^ cell number was found between unimpaired and impaired aged monkeys as classified by either task (Fig. 6*B*,*C*). Cholinergic neuron volume did not differ between age groups, or among the cognitive subgroups of aged monkeys, across any parcellation of the basal forebrain (*p* > 0.05; Fig. 6*D–F*).

**Table 5.** Number of ChAT and GAD67 immunopositive cells in basal forebrain of young and aged behaviorally characterized monkeys

	Population estimates					
	Young	Aged	DR-AU	DR-AI	DNMS-AU	DNMS-AI
ChAT^+^ cells (*n*)	156,881	143,956	145,112	142,470	145,112	142,470
(SE; *n*)	(10,132; 8)	(5359.11; 16)	(7856; 7)	(7594; 9)	(7856; 9)	(7594; 7)
rostral ChAT^+^ cells	34,331	38,171	37,943	38,463	37,943	38,463
(SE; *n*)	(3688; 8)	(3059; 16)	(4990; 7)	(3279; 9)	(4990; 9)	(3279; 7)
Caudal ChAT^+^ cells	122,529	105,678*	107,168	103,764	107,168	103,764
(SE; *n*)	(7484; 8)	(3512; 16)	(5017; 7)	(5124; 9)	(5017; 9)	(5124; 7)
GAD67^+^ cells	223,583	1,427,956*	134,716*	149,865*	153,827*	130,189*
(SE; *n*)	(22,858; 7)	(9517; 15)	(11448; 7)	(15,036; 8)	(14,770; 8)	(10,599; 7)
Rostral GAD67^+^ cells	89,271	56,654*	53,453*	59,454	61,338	67,032*
(SE; *n*)	(13,159; 7)	(3988; 15)	(5649; 7)	(5771; 8)	(6594; 8)	(3546; 7)
Caudal GAD67^+^ cells	134,312	85,770*	81,262	89,715	91,792	78,889*
(SE; *n*)	(19,784; 7)	(7638; 15)	(12,090; 7)	(10,215; 8)	(12,352; 8)	(8569; 7)

*p < 0.05 relative to young.

**Figure 7.**
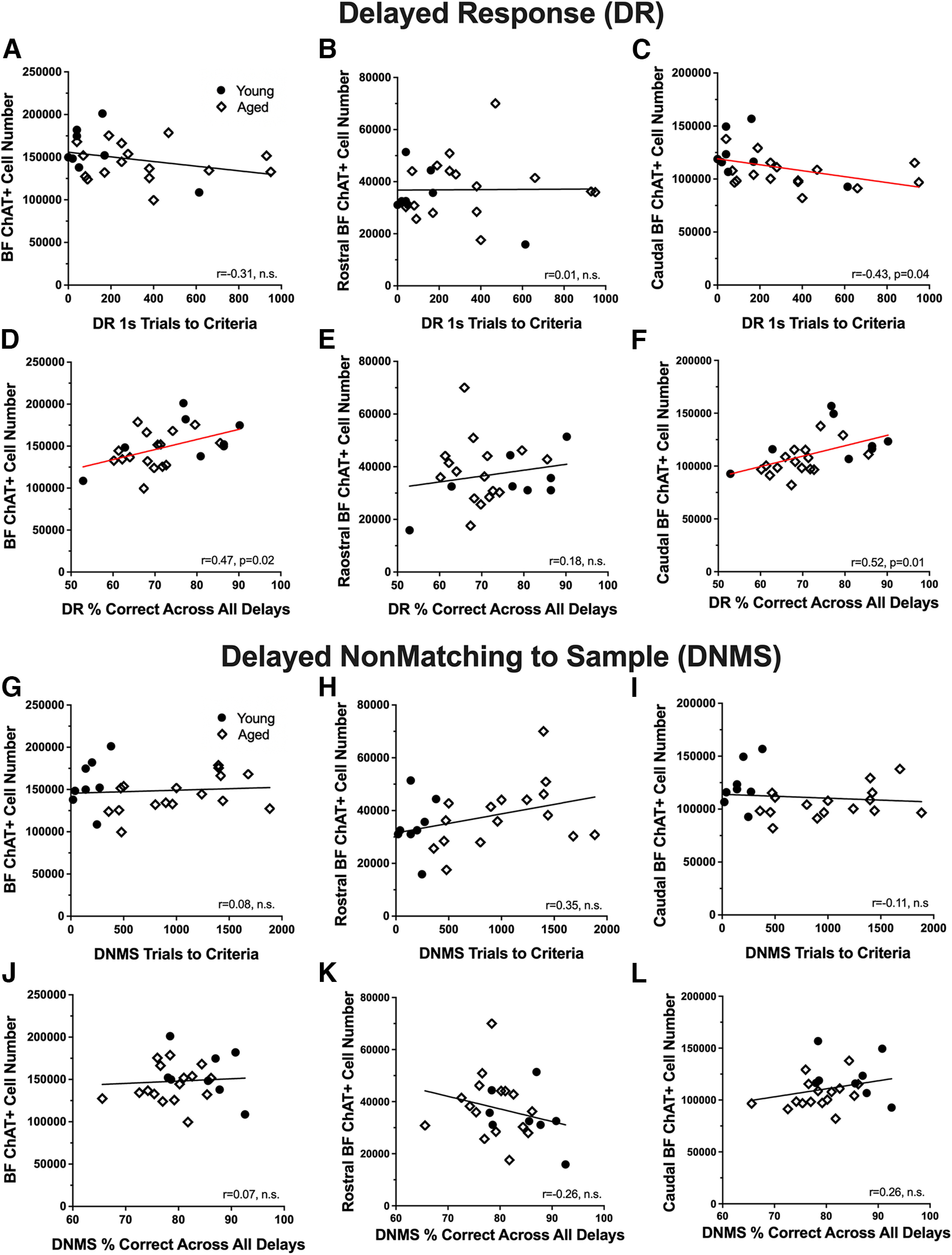
Relationship between ChAT immunopositive cell number and performance on behavioral tasks. ***A–C***, Correlations between ChAT^+^ cell numbers in the full extent of the basal forebrain (***A***), the rostral BF (***B***), the caudal BF (***C***), and the number of trials needed to reach criterion after a 1 s delay during the acquisition phase of the delayed response task (DR). ***D–F***, Scatter plots illustrating the relationship between ChAT^+^ cell numbers in the full extent of the basal forebrain (***D***), the rostral BF (***E***), the caudal BF (***F***), and the average percentage of correct choices across all delays during the testing phase of the DR task. ***G–I***, Correlations between ChAT^+^ cell numbers in the full extent of the basal forebrain (***G***), the rostral BF (***H***), the caudal BF (***I***), and the number of trials needed to reach criteria during the acquisition phase of the nonmatch to sample task (DNMS). ***J–L***, Relationships between ChAT^+^ cell numbers in the full extent of the basal forebrain (***J***), the rostral BF (***K***), the caudal BF (***L***), and the mean percentage of correct choices across all delays during the testing portion of the DNMS task. Red regression lines represent significant relationships, *p* < 0.05.

Following the same analytic strategy as for total BF number and size, the relationship between these morphometric parameters and behavioral performance was explored specifically for ChAT^+^ cells, using Pearson's *r* correlations. Cholinergic cell number in the caudal nuclei was significantly and negatively associated with DR acquisition such that monkeys with fewer cells required more trials to reach criterion (*r* = 0.43, *p* = 0.04; Fig. 7*C*). There was a significant positive relationship between DR delay performance and cholinergic cell number in both the full extent of the BF and the caudal divisions [BF: *r* = 0.47, *p* = 0.02 (Fig. 7*D*); caudal divisions: *r* = 0.52, *p* = 0.01 (Fig. 7*F*)]. As shown in Fig. 7*G–L*, there was no significant relationship between cholinergic cell number and either DNMS acquisition or recognition accuracy across delays. No significant correlation between cholinergic cell volume and any performance measure on either the DR or DNMS task was found (data not shown).

### GABAergic neuron number is prominently affected in the aged monkey basal forebrain

**Figure 8.**
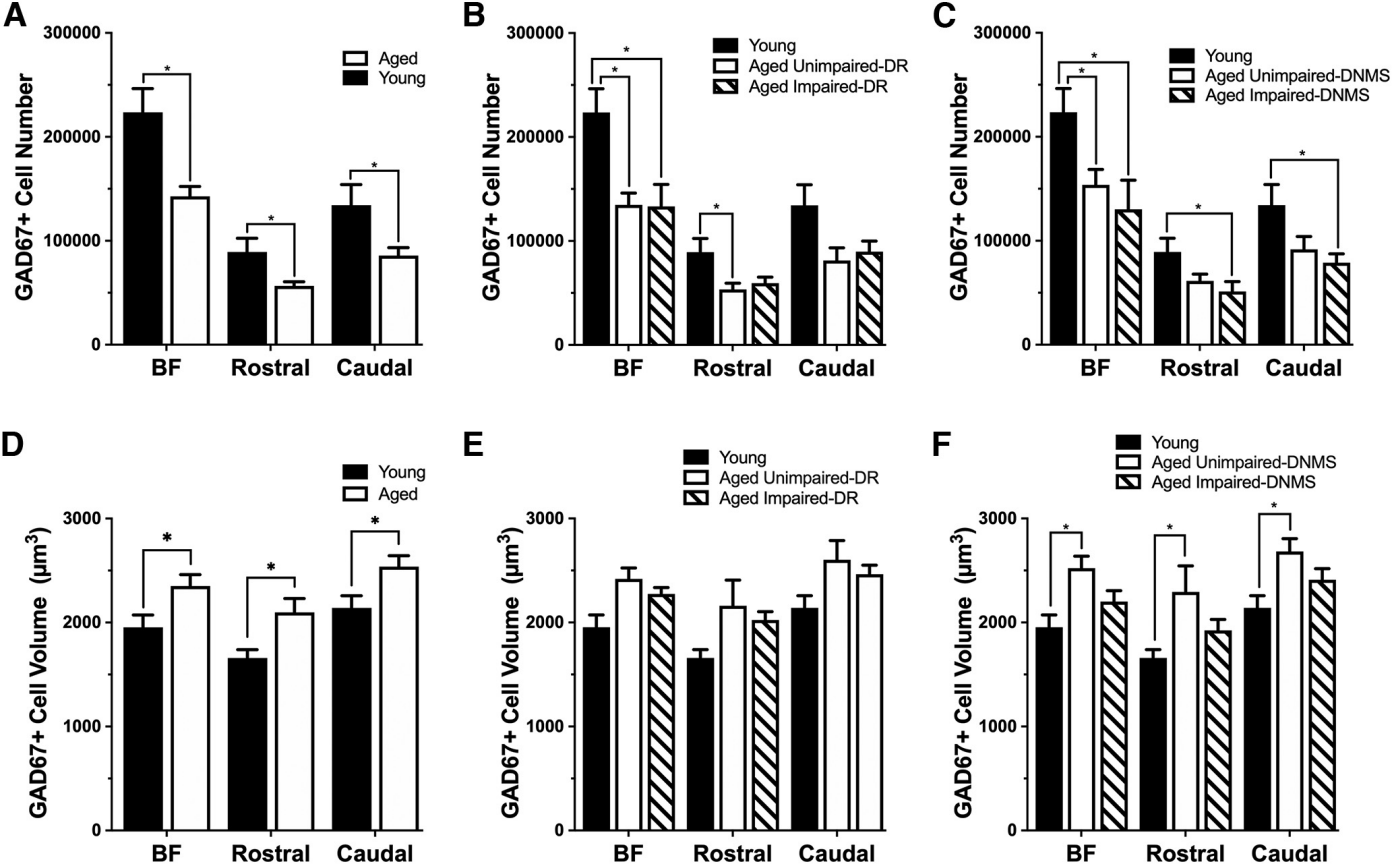
Mean (±SE) GABAergic (GAD67 immunopositive) cell number and volume in the entire basal forebrain, rostral BF, and the caudal BF in young (*n* = 8) and aged (*n* = 16) behaviorally characterized monkeys. ***A***, GABAergic cell numbers in the entire basal forebrain, the rostral BF, and the caudal BF were significantly less in aged monkeys compared with young monkeys. This significant difference was observed in both aged subgroups characterized on the delayed response task (***B***) and the delayed nonmatch-to-sample performance (***C***). ***D***, GABAergic cell volume was significantly increased in aged monkeys compared with young monkeys. ***E***, No significant difference was found between monkeys grouped based on performance in the delayed response task. ***F***, GABAergic cell volume was significantly increased in aged monkeys categorized as unimpaired in the delayed non-match-to-sample task. *p < 0.05.

GAD67 immunopositive cell number was significantly lower across the full extent of the basal forebrain in aged monkeys compared to young monkeys (unpaired *t* test: *t*_(20)_ = 3.90, *p* = 0.001; Fig. 8*A*, Table 5. The difference between age groups was significant in both the rostral and the caudal divisions (rostral nuclei, unpaired *t* test: *t*_(20)_ = 3.093, *p* = 0.006; caudal nuclei, unpaired *t* test: *t*_(20)_ = 2.800, *p* = 0.011; Fig. 8*A*). A comparison of GABAergic cell number between young monkeys and aged animals classified on the basis of DR and DNMS task performance revealed a highly significant group effect in the full basal forebrain as well as for the rostral and caudal divisions (one-way ANOVA; DR task main group effects, BF: *F*_(2,19)_ = 7.581, *p* = 0.004; rostral division: *F*_(2,19)_ = 4.726, *p* = 0.022; caudal division: *F*_(2,19)_ = 3.848, *p* = 0.040; DNMS task main group effects, BF: *F*_(2,19)_ = 8.121, *p* = 0.003; rostral division: *F*_(2,19)_ = 5.062, *p* = 0.017; caudal division: *F*_(2,19)_ = 4.017, *p* = 0.035; Fig. 8*B*,*C*). As observed for the cholinergic component of the basal forebrain, no significant difference in the number of GAD67 immunopositive cells was found between aged monkeys characterized as impaired and unimpaired on either task (*p* > 0.05; Fig. 8*D–F*).

### GABAergic cell volume is selectively greater in aged monkeys with intact visual recognition memory

In contrast to the stability seen among ChAT^+^ populations, the volume of GAD67 immunopositive cells in all divisions of the basal forebrain (i.e., full extent, rostral, and caudal) was significantly greater in aged monkeys compared to young monkeys (unpaired *t* test: BF: *t*_(20)_ = −2.188, *p* = 0.041; rostral division: *t*_(20)_ = −2.131, *p* = 0.046; caudal division: *t*_(20)_ = −2.277, *p* = 0.034; Fig. 8*D*, Table 6). GABAergic cell volume did not significantly differ among young monkeys and aged animals classified according to their DR performance (Fig. 8*E*). However, a comparison of the data in aged monkeys grouped on the basis of visual recognition memory revealed a highly significant difference among groups (one-way ANOVA: BF: *F*_(2,19)_ = 3.925, *p* = 0.037; rostral division: *F*_(2,19)_ = 3.861, *p* = 0.039; caudal division: *F*_(2,19)_ = 3.715, *p* = 0.043; Fig. 8*F*). *Post hoc* comparisons confirmed a significant increase in the volume of basal forebrain GAD67 immunopositive cells specifically in aged unimpaired monkeys relative to young across all regions (Scheffé's *post hoc* test; BF: Y vs AU, *p* = 0.038; Y vs AI, *p* = 0.471; rostral division: Y vs AU, *p* = 0.040; Y vs AI, *p* = 0.506; caudal division: Y vs AU, *p* = 0.043; Y vs AI, *p* = 0.391; Fig. 8*F*). There was no significant difference between the volume of GABAergic cells in aged behavioral subgroups, and, numerically, cell volume in the aged impaired monkeys appeared intermediate between values for young and aged unimpaired monkeys. Notably, the hypertrophy documented in GAD67 immunopositive neurons was neurochemically specific and not observed among ChAT immunopositive cells in adjacent histologic sections from the same brains. The effect was also selectively observed in aged monkeys with intact recognition memory compared to young adults, not in monkeys of the same advanced chronological age with impaired memory. Together with design safeguards ensuring all material was processed identically (for details, see Materials and Methods), the results count strongly against the potential contribution of a technical artifact.

**Table 6.** Volume of ChAT and GAD67 immunopositive cells in basal forebrain of young and aged behaviorally characterized monkeys

	Cell volume (µm^3^)					
	Young	Aged	DR-AU	DR-AI	DNMS-AU	DNMS-AI
ChAT^+^ cells	6732	7155	7115	7186	6959	7407
(SE; *n*)	(459; 8)	(371; 16)	(499; 7)	(559; 9)	(506; 9)	(572; 7)
Rostral ChAT^+^ cells	5739	5890	5838	5931	5785	6026
(SE; *n*)	(700; 8)	(391; 16)	(426; 7)	(635; 9)	(532; 9)	(619; 7)
Caudal ChAT^+^ cells	6998	7691	7607	7757	7471	7974
(SE; *n*)	(407; 8)	(386; 16)	(610; 7)	(529; 9)	(544; 9)	(567; 7)
GAD67^+^ cells	1953	2351*	2419	2274	2523*	2201
(SE; *n*)	(119; 7)	(110; 15)	(203; 7)	(61; 8)	(192; 8)	(106; 7)
Rostral GAD67^+^ cells	1659	2097*	2161	2024	2295*	1924
(SE; *n*)	(81; 7)	(134; 15)	(247; 7)	(80; 8)	(249; 8)	(106; 7)
Caudal GAD67^+^ cells	2141	2538*	2602	2463	2682*	2411
(SE; *n*)	(115; 7)	(105; 15)	(185; 7)	(88; 8)	(169; 8)	(169; 7)

*p < 0.05 relative to young.

**Figure 9.**
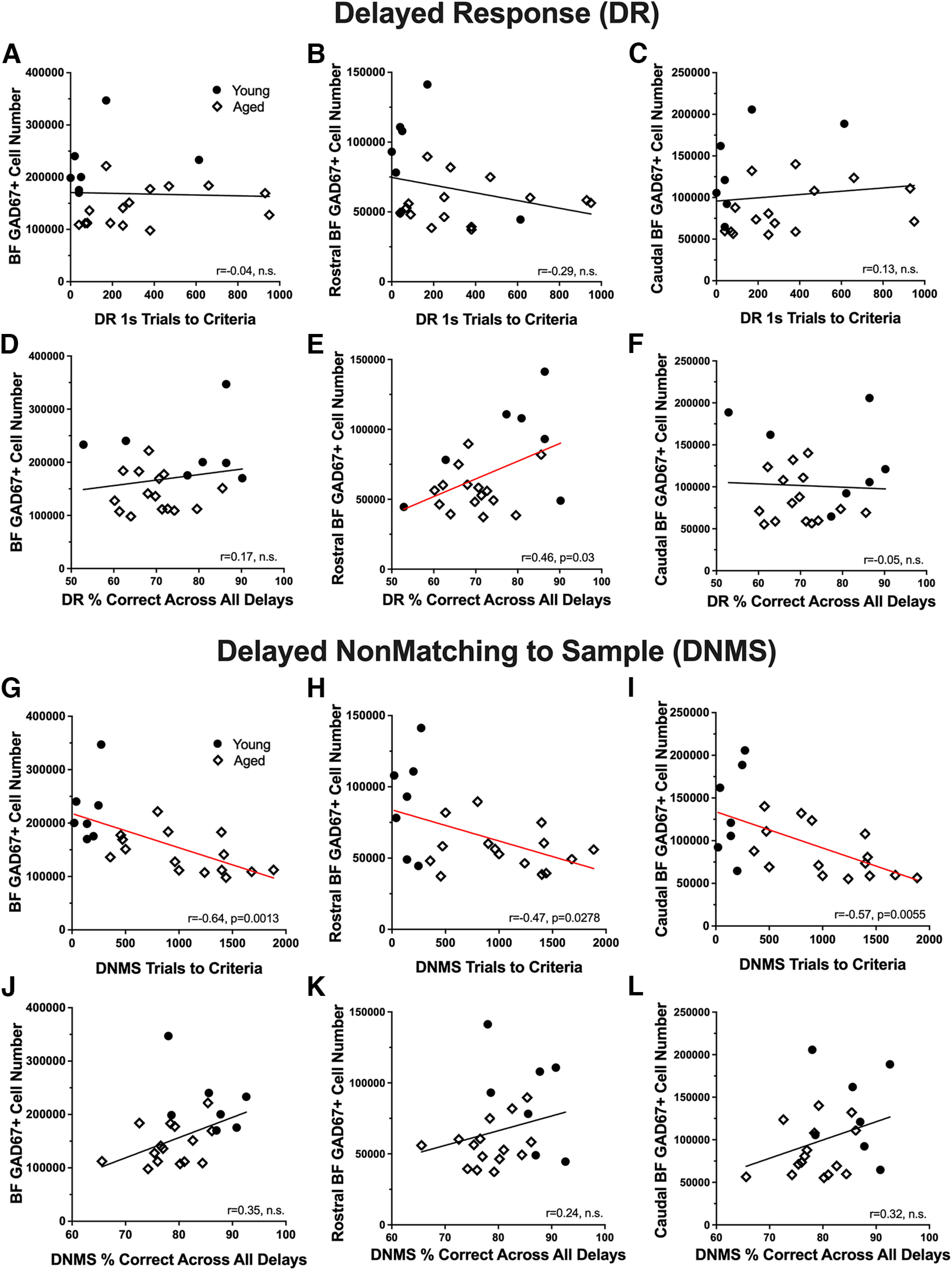
Relationship between GAD67 immunopositive cell number and performance on behavioral tasks. ***A–C***, Correlations between GAD67^+^ cell numbers in the full extent of the basal forebrain (***A***), the rostral BF (***B***), the caudal BF (***C***), and the number of trials needed to reach criterion after a 1 s delay during the acquisition phase of the delayed response task (DR). ***D–F***, Scatter plots illustrating the relationship between GAD67^+^ cell numbers in the full extent of the basal forebrain (***D***), the rostral BF (***E***), the caudal BF (***F***), and the average percentage of correct choices across all delays during the testing phase of the DR task. ***G–I***, Correlations between GAD67^+^ cell numbers in the full extent of the basal forebrain (***G***), the rostral BF (***H***), the caudal BF (***I***), and the number of trials needed to reach criteria during the acquisition phase of the delayed nonmatch to sample task (DNMS). ***J–L***, Relationships between GAD67^+^ cell numbers in the full extent of the basal forebrain (***J***), the rostral BF (***K***), the caudal BF (***L***), and the mean percentage of correct choices across all delays during the testing portion of the DNMS task. Red regression lines represent significant relationships, *p* < 0.05.

There was no significant relationship between GABAergic cell number and acquisition scores on the DR task (Fig. 9*A–C*), whereas cell number in the rostral division was significantly correlated with DR delay performance (rostral nuclei: *r* = 0.46, *p* = 0.03; Fig. 9*E*). GABAergic cell number in the BF, rostral division, and caudal division was significantly and negatively correlated with learning scores on the DNMS procedure such that monkeys with fewer cells took longer to reach criterion (BF: *r* = −0.64, *p* = 0.001; rostral nuclei: *r* = −0.47, *p* = 0.028; caudal nuclei: *r* = −0.57, *p* = 0.006; Fig. 9*G*–*I*). Correlation coefficients for DNMS delay performance with GABAergic cell number were all nonzero and positive, in the range *r* = 0.2–0.3, but none reached a statistically significant threshold (Fig. 9*J*–*L*). No significant correlations were observed between GABAergic cell volume and any measure of performance on the DR and DNMS tasks.

## Discussion

Our findings call for revision in long-standing perspectives on basal forebrain vulnerability in aging. Using a well-established model of cognitive aging in rhesus monkeys, the data accord with evidence that neuron number is significantly decreased in the aged primate BF relative to young adult values. These observations align with a theme dating back over 4 decades, encouraged initially by seminal reports that neuron dropout in the BF is an early feature of neurodegeneration in Alzheimer's disease (Whitehouse et al., 1982). Attention in this influential body of work has focused principally on the cholinergic neurons of the BF, owing partly to their prominent anatomic connectivity with widespread cortical sites that are required for normal cognitive function (Bartus et al., 1982; Whitehouse et al., 1982; Mesulam et al., 1983; Mann et al., 1984), together with evidence that anticholinergic pharmacological treatments induce marked memory impairment in normal subjects, mimicking geriatric memory dysfunction and features of dementia (Drachman and Leavitt, 1974). Indeed, the emphasis on this neurochemical subtype of cortically projecting BF cells has been so predominant that MRI reports of aging or AD-related changes in BF volume are often interpreted as synonymous with effects on the cholinergic system (Schmitz and Nathan Spreng, 2016; Butler et al., 2018). It has been known for many years, however, that the BF is neurochemically heterogeneous and that the same regions that give rise to corticopetal cholinergic projections are also resident to large populations of both locally and cortically projecting GABAergic neurons (Mesulam et al., 1983; Walker et al., 1989; Gritti et al., 2003). Evidence in rodents indicates that the basal forebrain also includes a significant population of glutamatergic neurons, which were not examined in the current study (Colom et al., 2005; Manseau et al., 2005; Henny and Jones, 2008). While the full diversity of inhibitory subpopulations remains to be examined, the novel insight reported here is that the frequently overlooked noncholinergic GAD67^+^ division of the primate BF is disproportionately vulnerable to aging, specifically in relation to cognitive outcome. Whether the observed changes in neuronal number represent frank neuronal loss or reduced GAD67^+^ expression is unknown and merits further study. Below we discuss the significant implications for the overall functional organization of the BF, neurocognitive aging and pathogenesis in neurodegenerative disease, and the development of therapeutic strategies.

Noncholinergic neurons in the monkey BF were initially described in research centered on the functional organization of cholinergic BF projections (Mesulam et al., 1983). A subset of these cells was subsequently identified as GABAergic, intermingled among cholinergic cells throughout the BF magnocellular complex (Walker et al., 1989). In all species where it has been examined, including mice, rats, and cats, BF GABAergic neurons include projection neurons that innervate widespread areas of the brain important for learning and memory, including the hippocampus and prefrontal cortex (Amaral and Kurz, 1985; Fisher et al., 1988; Linke et al., 1994; Gritti et al., 2003). While BF GABAergic cortical projections have not been similarly verified in monkeys, early retrograde tracer studies indicate that substantial numbers of cortically projecting BF neurons in the monkey are noncholinergic (Mesulam et al., 1983). The available comparative evidence is therefore strongly suggestive of a GABAergic contribution to BF corticopetal projections in primates. Here we confirm and extend earlier findings in rats (Gritti et al., 2006), demonstrating that GABAergic neurons in the primate BF significantly outnumber the cholinergic component. Capitalizing on a unique sample of behaviorally characterized aged monkeys in a large quantitative morphometric analysis of this cell group, the results document substantial neuronal dropout throughout all divisions of the BF compared to younger adult values. The volume of GABAergic cells was also affected, with the most robust effect consisting of hypertrophy in aged animals that displayed relatively preserved visual recognition memory. Age differences in cholinergic cell groups, by comparison, were anatomically restricted to posterior divisions of the BF, and neuron size was preserved throughout. While the net functional significance of these regionally selective effects may well be substantial, our findings document that the impact of aging on the primate BF extends beyond the cholinergic system and prominently involves cells positioned to provide extensive local and cortical inhibitory drive.

**Figure 10.**
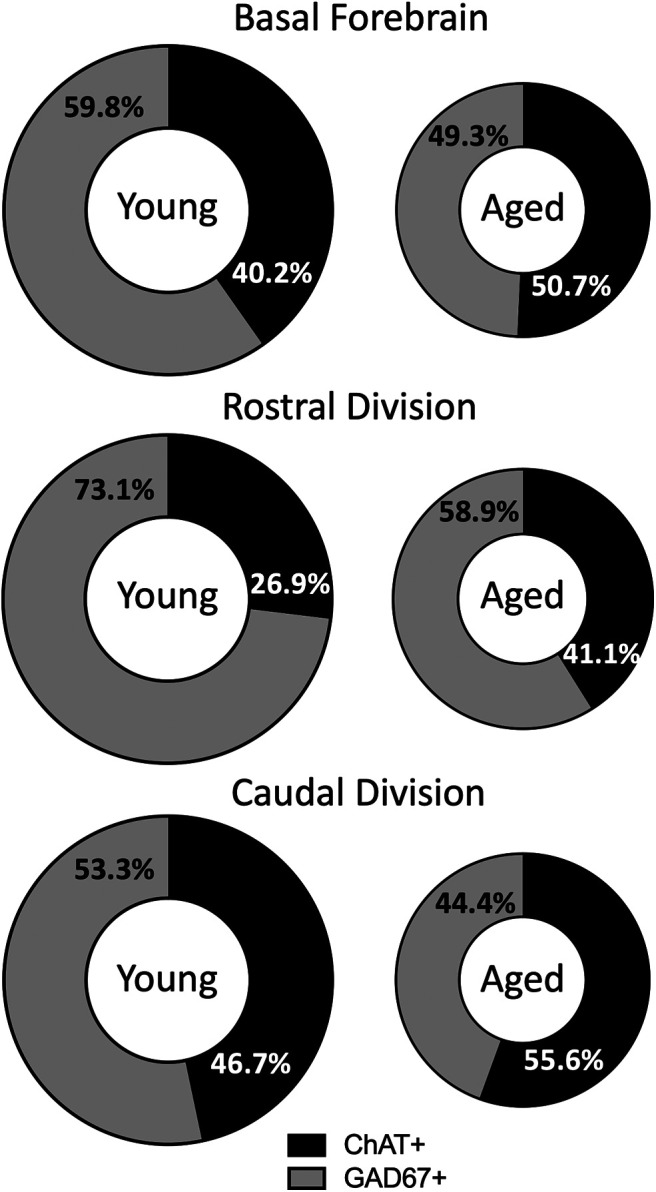
Percentage of total BF comprised of ChAT and GAD67 immunopositive neurons. Donut graphs represent the total BF according to the percentage of cells that are ChAT^+^ or GAD67^+^. Within each anatomical division, the donut size for the aged monkeys represents the total cell number relative to the young monkeys. The black portion of the donut represents the proportion of BF neurons that are excitatory cholinergic neurons, and the gray portion represents the proportion of BF neurons that are inhibitory GABAergic neurons.

Considered together, the neurochemical heterogeneity of aging effects reported here implies substantial BF reorganization throughout its widespread sphere of influence. Figure 10 illustrates that the age-related differences in cell number we observed comprise a significant shift in the balance of inhibitory potential in the BF. In young monkeys, GABAergic neurons accounted for just under 60% of the total counted, with presumptive excitatory cholinergic neurons comprising the remaining 40%. Relative to this inhibitory-predominant organization, aging appears to result in a shift toward excitatory potential, with an approximately equal representation of cholinergic and GABAergic neurons. Although the precise percentages varied across the rostrocaudal extent of the BF, an age-related increase favoring a cholinergic influence was apparent in all divisions, with shifts on the order of 10–15%. Whether the increase in GABAergic cell volume we found in aged monkeys with preserved recognition memory reflects a compensatory response to the concurrent shift toward excitatory drive merits investigation. Overall, our findings highlight that a full account of BF contributions to cognitive aging will require a fresh perspective that extends beyond the cholinergic system.

Changes in E/I balance are prominently implicated in both aging and the pathogenesis of neurodegenerative disease, including compelling reports of elevated neuronal activity in association with age-related cognitive impairment in rodent models, monkeys, and humans (Luebke et al., 2004; Bories et al., 2013; Thomé et al., 2016; Baker et al., 2019). Conversely, pharmacological treatments that blunt neuroimaging signatures of excess activity can improve cognitive function in both MCI patients and aged rats (Koh et al., 2010; Bakker et al., 2012). Both amyloid deposition and tau propagation are known to be neuronal activity-dependent, potentially directly linking shifts in E/I balance to the pathologic cascade and progression of AD (Liu et al., 2012; Wu et al., 2016). Whether these observations, together with the findings reported here, are related to evidence that basal forebrain degeneration precedes and predicts the cortical spread of pathology found in Alzheimer's disease (Schmitz and Nathan Spreng, 2016), warrants exploration. However, it should be noted in this context that the functional consequence of age-related shifts in neuronal activity will critically depend on how local and distal projection networks are affected. Whereas long-projecting GABAergic BF cells may preferentially target cortical inhibitory interneurons, locally acting GABAergic cells synapse on other BF populations, including corticopetal cholinergic and GABAergic neurons, and in this way can influence both cholinergic and GABAergic innervation of the hippocampus and cortex (Záborszky et al., 1986; Smiley and Mesulam, 1999; Gritti et al., 2003). The net functional result of these interactions will also depend on the receptor subtype profiles of recipient cells, including resident variants of both cholinergic and GABAergic receptors. The influence of the prominent yet understudied glutamatergic component of the basal forebrain, as well as other neuropeptide cell populations, is also unknown. Recent neurophysiological data underscore the need for further research into the influence of neurochemically specific cell types on the functional organization of the primate BF (Zhang et al., 2019). Our analysis further highlights the need to explore BF cellular diversity and determine the circuit**-**specific details of the changes we document.

In conclusion, our findings call for significant reconceptualization of BF vulnerability in aging. While we confirm that the primate BF is prominently affected, the evidence indicates that this vulnerability reflects prominent changes involving the noncholinergic BF, resulting in a significant shift in the relative balance of neurochemically specific subpopulations. These include effects associated with both age-related cognitive impairment (i.e., decreases in GABAergic immunopositive cell number throughout the rostrocaudal extent of the BF) and, notably, other morphometric alterations that may comprise compensatory responses supporting successful cognitive outcomes (i.e., increases in GABAergic positive cell volume in aged monkeys with preserved recognition memory). The latter findings encourage increased attention to inhibitory neuronal populations as a promising novel pharmacological target for healthy cognitive aging.
